# The Zebrafish Antiapoptotic Protein BIRC2 Promotes *Edwardsiella piscicida* Infection by Inhibiting Caspases and Accumulating p53 in a p53 Transcription-Dependent and -Independent Manner

**DOI:** 10.3389/fimmu.2021.781680

**Published:** 2021-11-23

**Authors:** Lu Cao, Dong Yan, Jun Xiao, Hao Feng, Ming Xian Chang

**Affiliations:** ^1^ State Key Laboratory of Freshwater Ecology and Biotechnology, Institute of Hydrobiology, Chinese Academy of Sciences, Wuhan, China; ^2^ College of Advanced Agricultural Sciences, University of Chinese Academy of Sciences, Beijing, China; ^3^ State Key Laboratory of Developmental Biology of Freshwater Fish, College of Life Science, Hunan Normal University, Changsha, China; ^4^ Innovation Academy for Seed Design, Chinese Academy of Sciences, Wuhan, China

**Keywords:** antiapoptotic protein BIRC2, caspases, p53, negative regulation, *Edwardsiella piscicida* infection

## Abstract

IAPs (inhibitors of apoptosis) are endogenous caspase inhibitors with multiple biological activities. In the present study, we show functional characteristics of antiapoptotic protein BIRC2 (cIAP1) in response to *Edwardsiella piscicida* infection. Overexpression of BIRC2 in zebrafish larvae promoted the proliferation of *E. piscicida*, leading to a decreased larvae survival. The expression levels of caspases including *casp3*, *casp8*, and *casp9* were significantly inhibited by BIRC2 overexpression in the case of *E. piscicida* infection. Treatment of zebrafish larvae microinjected with BIRC2 with the caspase activator PAC-1 completely blocked the negative regulation of BIRC2 on the *E. piscicida* infection, with the reduced inhibition on the *casp3* and without inhibition on *casp8* and *casp9*. In contrast to the regulation of BIRC2 on the caspases, BIRC2 overexpression significantly induced the expression of p53, especially at 24 hpi. In addition to the cytoplasmic p53 expression, BIRC2 overexpression also induced the expression of the nuclear p53 protein. Further analysis demonstrated that BIRC2 could interact and colocalize with p53 in the cytoplasm. The numbers of *E. piscicida* in larvae overexpressed with BIRC2 and treated with pifithrin-μ (an inhibitor of mitochondrial p53) or pifithrin-α (an inhibitor of p53 transactivation) were lower than those of larvae without pifithrin-μ or pifithrin-α treatment. Critically, the p53 inactivators pifithrin-μ and pifithrin-α had no significant effect on larval survival, but completely rescued larval survival for zebrafish microinjected with BIRC2 in the case of *E. piscicida* infection. Collectively, the present study suggest that piscine BIRC2 is a negative regulator for antibacterial immune response in response to the *E. piscicida* infection *via* inhibiting caspases, and accumulating p53 in a p53 transcription-dependent and -independent manner.

## Highlights

1. Overexpression of zebrafish BIRC2 promotes the proliferation of *E. piscicida*.
2. Overexpression of zebrafish BIRC2 regulates the transcriptions of caspases and p53.
3. Zebrafish BIRC2 interacts and colocalizes with p53 in the cytoplasm.
4. The caspase activator PAC-1 blocks the negative regulation of zebrafish BIRC2.
5. The p53 inactivators pifithrin-μ and pifithrin-α block the negative regulation of zebrafish BIRC2.

## Introduction

The inhibitor of apoptosis proteins (IAPs), also known as baculovirus IAP repeat (BIR)-containing proteins (BIRCs), are a family of proteins that are evolutionarily conserved in plants, animals and microorganisms (1–3). In mammals, eight members including BIRC1/NAIP, BIRC2/cIAP1, BIRC3/cIAP2, BIRC4/XIAP, BIRC5/Survivin, BIRC6/Apollon, BIRC7/ML-IAP, and BIRC8/ILP2 have been identified. Among them, BIRC2, BIRC3, and BIRC4, which own three tandem BIR domains, one ubiquitin associated (UBA) domain and one C-term RING domain, can bind caspases and IAP antagonists through the BIR domains (4). However different from BIRC4, while BIRC2 and BIRC3 bind to caspase-3, caspase-7, and caspase-9, they do not inhibit proteolytic activities of caspases (5, 6). Instead, BIRC2 has been suggested to neutralize caspases by promoting their proteasomal degradation (6). BIRC2 and BIRC3 were also shown to protect cells from apoptosis by acting as E3 ubiquitin ligases for effector caspases and receptor-interacting protein 1 (RIP1), whose E3 ligase activity was conferred by their C-terminal RING domain (6, 7). In addition, BIRC2 and BIRC3 contain a central caspase-associated recruitment domain (CARD). A functional nuclear export signal has been identified in the CARD domain of BIRC2, and the nucleocytoplasmic redistribution of BIRC2 is observed in human monocytes as well as in tumor cells (8). Furthermore, the E3 ligase activity of BIRC2 mediated by the RING domain is inhibited by the CARD of BIRC2 (9).

It is now clear that BIRC2, BIRC3, and BIRC4 have a much wider spectrum of action. These IAPs regulate innate immunity and inflammatory responses by promoting ubiquitylation not only downstream of various PRRs including NOD1/2, Toll-like receptors (TLRs) and RIG-I signaling to regulate Nuclear Factor-κB (NF-κB), mitogen-activated protein kinase (MAPK) or IRF pathways, but in the signaling cascades activated by pro-inflammatory cytokines such as TNF to regulate MAPK, canonical and non-canonical NF-κB signaling (10). The prosurvival function of BIRC2 and BIRC3 is involved in their ability to activate the canonical NF-κB pathway, which drives expression of various prosurvival molecules (11). BIRC2 and BIRC3 are important components of the TNFα-mediated NF-κB signaling pathway, and either BIRC2 or BIRC3 is required to protect cells against TNFα-induced apoptosis (12). Furthermore, BIRC2 and BIRC3 protect cells from death by regulating RIP1 ubiquitination (13) and preventing RIP1/RIP3-dependent ROS production (14). During pathogenic infection, BIRC2 and BIRC3 promote host survival by preventing death-receptor-induced programmed necrosis in immune and non-immune cells. For example, mice with the BIRC3 deficiency exhibited the increased susceptibility and mortality to influenza A virus infection, and BIRC3-dependent antagonism of RIP3-mediated programmed necrosis protected the mice from influenza infection by maintaining pulmonary tissue homeostasis (15). BIRC2 or BIRC3 deficiency increased macrophage necrosis and decreased control of the intracellular bacterium *Listeria monocytogenes* (16).

Besides caspases and IAPs, the p53 tumor suppressor protein is also involved in the control of apoptosis. A study showed that BIRC2 was involved in p53-induced apoptosis. This cleavage of BIRC2 by serine protease(s) was p53-dependent but caspase-independent, and was required for apoptosis (17). Many findings also revealed the clinical relevance between IAPs and p53. For instance, p53 has been reported to be a key downstream effector of BIRC6, and the interaction between BIRC6 and p53 facilitated the degradation of p53, which led to carcinogenesis and an anti-apoptotic status (18). Here we report that overexpression of piscine BIRC2 in zebrafish larvae leads to the increased susceptibility and mortality in response to *E. piscicida* infection. Compared with wild-type (WT) zebrafish, the bacterial loads were higher in zebrafish larvae overexpressed with BIRC2. We also demonstrate that piscine BIRC2 regulates the transcription of caspases and p53. Furthermore, the suppression of caspases and accumulation of cytoplasmic and nuclear p53 have been shown to be crucial for negative regulation of BIRC2 during bacterial infection. These data may provide clues for understanding the functions of apoptosis-related protein in pathogen infection and the underlying mechanisms of piscine BIRC2 in the negative regulation of bacterial infection.

## Materials and Methods

### Ethics Statement

All animal experiments were conducted in accordance with the Guiding Principles for the Care and Use of Laboratory Animals and were approved by the Institute of Hydrobiology, Chinese Academy of Sciences (Approval ID: IHB 2013724).

### Zebrafish, Cell Lines, and Bacteria

The wild-type AB/TU zebrafish were maintained in recirculating aquaculture system at 28.5°C under standard conditions according to zebrafish care and maintenance protocol. Epithelioma papulosum cyprini (EPC) cells were maintained at 28°C in 5% CO_2_ in medium 199 (Hyclone) supplemented with 10% FBS, 100 U/ml penicillin and 100 μg/ml streptomycin. The wild-type *Edwardsiella piscicida* (PPD130/91 strain) were grown in tryptic soy broth (TSB, BD Biosciences) (19).

### Plasmid Construction and Sequence Analysis

Based on zebrafish mRNA sequence (GenBank accession No: NM_194395), primers FLAG-BIRC2F/FLAG-BIRC2R were used for cloning the open reading frame (ORF) of zebrafish BIRC2, and inserted into the p3×FLAG-CMV ™-14 Expression Vector (Sigma-Aldrich). The primer sequences used for plasmid construction were listed in **Table 1**.

**Table 1:** Primer information.

Name	Sequence	Application
FLAG-BIRC2F	CCGGAATTCATGGAAATATTACAAAACAG	Ligated to p3xFLAG-CMV™-14 vector
FLAG-BIRC2R	CGCGGATCCAGAGAGGAAAGTACGGACAG	
p53F	CTCAGGTTCCCGCAGTC	Quantitative real-time PCR
p53R	TCCATTCAGCACCAAGC	
BIRC2F	CTTACCTGCCACCGCAAACCTCC	
BIRC2R	CGTTCCTGTTCCCGACGCATACC	
casp8F	GTGTCTGTTGACGAAATACGA	
casp8R	GTGACTGAATAAACCAGGAGC	
casp3aF	TGTTCTTTATTCAGGCTTGTCG	
casp3aR	CTGCCATACTTTGTCATCATTT	
casp3bF	ACGGTGTAGGTGACGAGGAAAC	
casp3bR	AGGAGATAAACCAGGAGCCATT	
casp9F	AGGCATTGAATCCCGAAGA	
casp9R	ACAGGAGGGCGATGAACAC	
NF-κB1F	TGTGGTTCGGCTGATGTTC	
NF-κB1R	GGTTCGCTCGTCTCGTTGT	
NF-κB2F	AAGATGAGAACGGAGACACGC	
NF-κB2R	TCTACCAGCAATCGCAAACAA	

Domain architecture analysis was performed using Batch CD-Search (NCBI), and search results were visualized through the TBtools (20). Construction of phylogenetic tree was performed using the Neighbor-Joining method with 10,000 bootstrap replications using MEGA X software. Accession numbers of BIRC2 proteins from different species used are as follows: zebrafish (*Danio rerio*) BIRC2, XP_005161213.1; common carp (*Cyprinus carpio*) BIRC2, XP_018950329; channel catfish (*Ictalurus punctatus*) BIRC2, NP_001187106.1; rainbow trout (*Oncorhynchus mykiss*) BIRC2, XP_036845322.1; Japanese medaka (*Oryzias latipes*) BIRC2, XP_023818594.1; Atlantic cod (*Gadus morhua*) BIRC2, XP_030216815.1; spotted gar (*Lepisosteus oculatus*) BIRC2, XP_006627852; human (*Homo sapiens*) BIRC2, NP_001157.1; house mouse (*Mus musculus*) BIRC2, NP_031491.2; chicken (*Gallus gallus*) BIRC2, NP_001007823.1; green anole (*Anolis carolinensis*) BIRC2, XP_016848156.1; and common frog (*Rana temporaria*) BIRC2, XP_040196143.1.

### Constitutive and Inducible Expression of Zebrafish BIRC2

Zebrafish embryos and larvae including 6 h post-fertilization (hpf), 12 hpf, 1 days post-fertilization (dpf), 2 dpf, 3 dpf, 4 dpf, 5 dpf and 7 dpf were collected, and used for quantivative RT-PCR (qRT-PCR) to determine the constitutive expression of zebrafish BIRC2. To analyze the expression level of zebrafish BIRC2 in response to bacterial infection, zebrafish larvae at 4 dpf were infected with *E. piscicida* (2 × 10^8^ CFU/ml) at 28°C. Zebrafish larvae were collected at 6, 24 and 72 h post-infection (hpi), and used for qRT-PCR. The primer sequences used for qRT-PCR were listed in **Table 1**.

### Apoptosis Analysis of Zebrafish BIRC2

To determine the effect of zebrafish BIRC2 on apoptosis, 1 × 10^6^ EPC cells were plated into 6-well plates overnight and then transiently transfected with 2 μg BIRC2-FLAG or p3×FLAG. After 24 h later, the cells were infected with *E. piscicida* at an MOI of 5, 10 or left untreated. At 6 hpi, these cells were washed with ice-cold PBS, and proceeded with the FITC Annexin V Staining using the FITC Annexin V Apoptosis Detection Kit (Vazyme, China) on a CytoFLEX LX Flow Cytometer (Beckman, USA) according to the manufacturer’s instructions. The data were analyzed using the software CytoExpert.

### Colony Counting, Survival Analysis, and Sample Collection

To determine the role of zebrafish BIRC2 in bacterial infection, 200 ng/μl p3×FLAG empty plasmid or BIRC2-FLAG was microinjected into zebrafish embryos at the one-cell stage. At 4 dpf, the hatched larvae were infected with 2 × 10^8^ CFU/ml *E. piscicida*. To determine whether the negative regulation of zebrafish BIRC2 on *E. piscicida* infection was related to the suppression of caspases and the accumulation of p53, the caspase activator PAC-1 (Selleck, #S2738) and p53 inactivators pifithrin-μ (Selleck, #S2930) and pifithrin-α (Selleck, #S2929) were used for treating zebrafish larvae microinjected with p3×FLAG or BIRC2-FLAG at 4 dpf. The final concentration of treatment is 10 μm for PAC-1, 50 μm for Pifithrin-μ, and 5 μm for Pifithrin-α. After 12 h treatment, these larvae were infected with 2 × 10^8^ CFU/ml *E. piscicida*.

At 24 and/or 48 hpi, 10 larvae per group were washed in PBS three times and then lysed in 1 ml of PBS *via* homogenization for plating serial dilutions on TSB agar. CFU of *E. piscicida* were enumerated after 24 h of incubation at 28°C. For larval survival analysis, exposures were performed with three repetitions for each group, and the numbers of each repetition were 20 larvae. The numbers of surviving larvae were counted daily for 4 or 5 d. GraphPad Prism 7 was used to generate survival curves. To determine the effect of zebrafish BIRC2 on the expression levels of apoptosis-related genes and NF-κB genes, 30 larvae per group were collected at 24 and 48 hpi, and used for qRT-PCR. To determine the effect of PAC-1 on the expression levels of apoptosis-related genes and NF-κB genes, 30 larvae per group were collected at 48 hpi, and used for qRT-PCR.

### qRT-PCR

For qRT-PCR analysis, total RNA was extracted from those samples collected above by Trizol reagent (Invitrogen). RNase-free DNase I (Thermo) was used to remove genomic DNA contamination at 37°C for 30 min. The first-strand cDNA was generated from total RNA using RevertAid First Strand cDNA Synthesis Kit (Thermo Fisher Scientific). qRT-PCR was performed on a BIO-RAD CFX96 Real-Time System using SYBR^®^ Green Master Mix (Bio-RAD) according to the procedure as follows: preincubation at 95°C for 3 min, then 45 cycles at 95°C for 10 s, 58°C for 20 s, and 72°C for 30 s. Each sample was tested in triplicate. The efficiency of primer pairs was calculated by making a dilution series. The Ct values were correlated with the DNA input over seven orders of magnitude (R^2^ >0.999). The amplification efficiency of *BIRC2*, *casp3a*, *casp3b*, *casp8*, *casp9*, *NF-κB1*, *NF-κB2*, *p53*, and *GAPDH* primer pairs is 90.0, 93.5, 93.4, 94.4, 92.7, 93.7, 92.9, 94.3, and 92.5%, respectively. The housekeeping gene GAPDH was used for normalizing the Ct values of target genes. The fold changes relative to control group were calculated using the 2^−ΔΔCt^ method. All primers used for qRT-PCR were presented in **Table 1**.

### Fluorescence Microscopy

Seeded on coverslips were 2 × 10^5^ EPC cells inside 24-well plates that were co-transfected with FLAG plus GFP, FLAG plus p53-GFP, BIRC2-FLAG plus p53-GFP, and then infected with *E. piscicida* at an MOI of 1. At 6 hpi, the cells were washed with PBS for three times, fixed with 4% formaldehyde at 37°C for 15 min, permeabilized with PBS containing 0.1% Triton^®^ X-100 for 10 min, incubated with anti-FLAG antibody (1:5,000, Sigma-Aldrich, # F3165) overnight, and incubated with ReadyProbes™ Alexa Fluor^®^ 594 Goat Anti-Mouse IgG Antibody (Invitrogen, #R37121) for 30 min. After washing with PBST, the cells were stained with NucBlue Fixed (Invitrogen, #R37606) and observed on a confocal microscope (SP8; Lecia, Wetzlar, Germany).

### Co-Immunoprecipitation and Western Blotting

To investigate the regulating effect of zebrafish BIRC2 on the p53 protein with or without bacterial infection, 1 × 10^6^ EPC cells were plated into 6-well plates, and transfected with indicated plasmids and concentration. After 48 h, these cells were infected with *E. piscicida* at an MOI of 1 or left untreated. At 6 hpi, these cells were used for protein extraction using RIPA buffer (Thermo Scientific™) containing Protease Inhibitor Cocktail. All samples were analyzed by Western blotting with anti-GAPDH (Proteintech, #10494-1-AP), anti-FLAG (Sigma-Aldrich, #F3165), and anti-pTurboGFP (Evrogen, #AB513) antibodies.

To investigate the exact effect of zebrafish BIRC2 on the protein regulation of cytoplasmic and nuclear p53 in the case of *E. piscicida* infection, 1 × 10^6^ EPC cells plated into 6-well plates were transfected with indicated plasmids and concentration. At 6 hpi, the cells were collected and used for subsequent nuclear and cytoplasmic protein extraction using Subcellular Protein Fractionation Kit (Thermo Scientific™, #78840) according to the manufacturer’s instructions. Extracts were analyzed by Western blotting with anti-HDAC1 (ab41407; Abcam), anti-tubulin (ab6046; Abcam), anti-FLAG (F3165; Sigma-Aldrich) and anti-pTurboGFP (AB513; Evrogen) antibodies.

To determine the interaction between zebrafish BIRC2 and p53, EPC cells were transfected with the indicated plasmids mixes (1,000 ng for each plasmid). After 48 h, the cells were collected and lysed in IP lysis buffer containing Protease Inhibitor. The Anti-FLAG M2-Agarose Affinity Gel (Sigma-Aldrich) was added to the cell lysates, incubated at 4 ˚C overnight with gentle mixing. The eluted proteins and input proteins were analyzed by Western blotting with anti-FLAG and anti-pTurboGFP antibodies.

### Statistical Analysis

All data were expressed as means and standard errors of means (SEM) of three independent experiments. A two-tailed Student’s T-test or a one-way ANOVA was used to determined significant differences for qRT-PCR (*p <0.05; ** *p <*0.01). The log-rank test was used to test significant differences for survival analysis.

## Results

### Sequence Features of Zebrafish BIRC2

**Figure 1:**
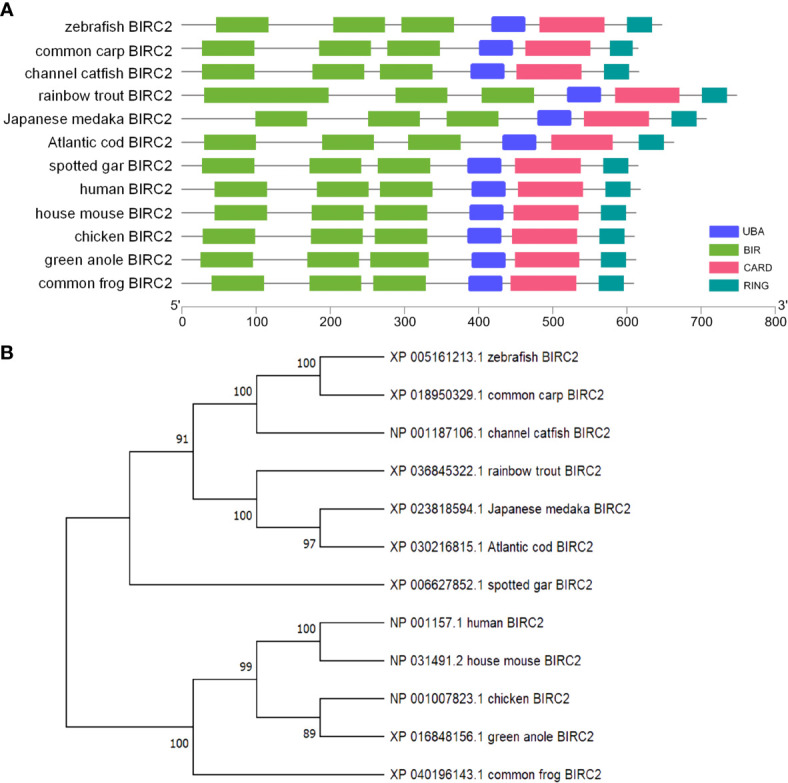
Bioinformatics analysis of zebrafish BIRC2. **(A)** Schematic diagram of the modular domain architecture of vertebrate BIRC2. **(B)** Phylogenetic tree of vertebrate BIRC2. The amino acid sequences are aligned using CLUSTALW, and the tree is constructed by the N-J method supported with 10,000 bootstrap replications using MEGA X software.

The ORF length of zebrafish BIRC2 is 1,887 bp (GenBank accession No. NM_194395), encoding 628 amino acids. Sequence analysis by using NCBI CDD reveals that vertebrate BIRC2 proteins from zebrafish, common carp, channel catfish, rainbow trout, Japanese medaka, Atlantic cod, spotted gar, frog, green anole, chicken, mouse, and human have the same domain architecture, and all proteins contain three BIR domains, one UBA domain, one CARD domain, and one C-term RING domain (**Figure 1A**). A phylogenetic tree is constructed using the N-J method based on the amino acid sequences from these vertebrate BIRC2 proteins. The results show that zebrafish BIRC2 is grouped with common carp and channel catfish BIRC2 proteins, with bootstrap value 100% (**Figure 1B**).

### Expression Analysis of Zebrafish BIRC2

**Figure 2:**
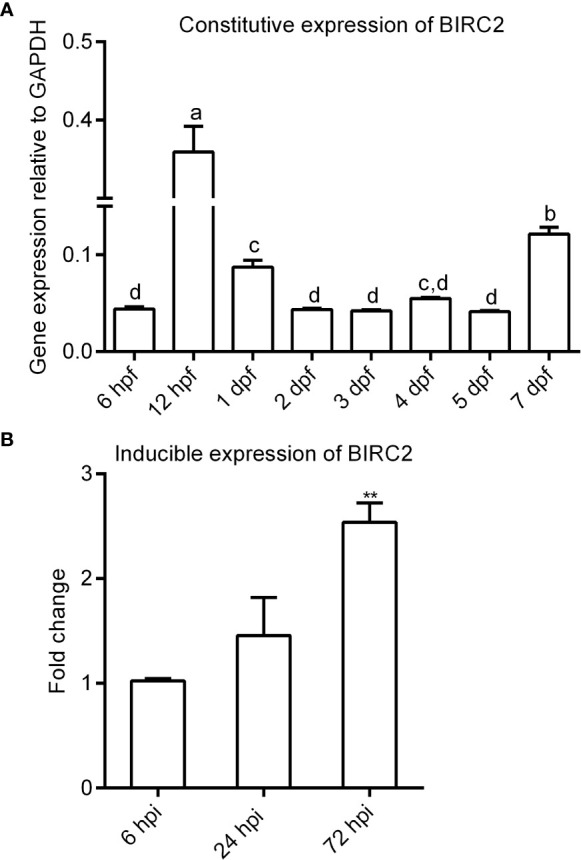
The expression patterns of zebrafish BIRC2. **(A)** The constitutive expression of zebrafish BIRC2 in zebrafish embryos and larvae. Means with different letters are statistical different. **(B)** The inducible expression of zebrafish BIRC2 in zebrafish larvae infected with *E. piscicida*. Data represented the means ± the SEM (n = 3) and were tested for statistical significance using a two-tailed student’s *t-*test or one-way ANOVA. **p < 0.01.

The expression of zebrafish BIRC2 was examined in eight samples involved in early developmental stages and in zebrafish larvae with or without *E. piscicida* infection. The results from qRT-PCR showed that the transcript level of zebrafish BIRC2 sharply increased from 6 to 12 hpf, then decreased from 12 hpf to 2 dpf, next maintained a relatively stable level from 2 to 5 dpf, and finally increased at 7 dpf (**Figure 2A**). Compared with the control group without *E. piscicida* infection, the expression of BIRC2 in zebrafish larvae infected with *E. piscicida* had no significant change at 6 and 24 hpi during the early stage of infection, but significantly increased at 72 hpi during the late stage of infection (**Figure 2B**).

### Anti-Apoptosis Activity of Zebrafish BIRC2

**Figure 3:**
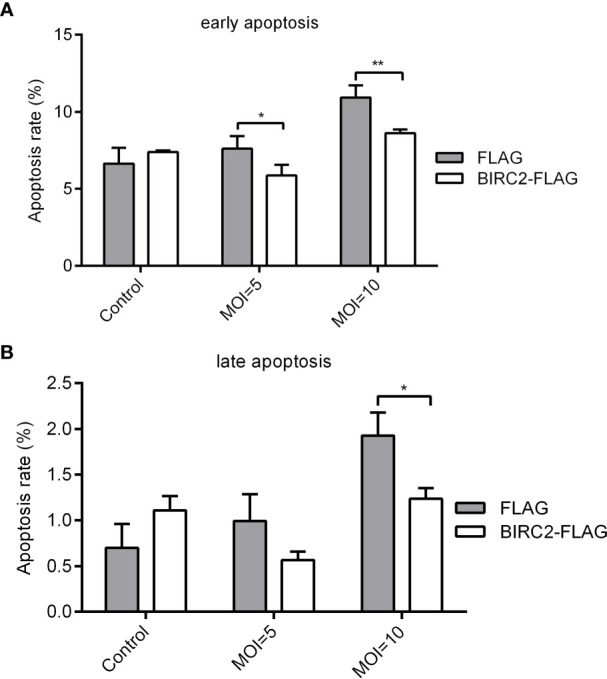
Overexpression of zebrafish BIRC2 inhibits the early and late apoptosis in the case of *E. piscicida* infection. **(A)** The effect of zebrafish BIRC2 on the early apoptosis. **(B)** The effect of zebrafish BIRC2 on the late apoptosis. The EPC cells transfected with p3×FLAG or BIRC2-FLAG were infected with *E. piscicida* at an MOI of 5, 10 or left untreated. At 6 hpi, samples were collected, then stained with annexin V FITC, and finally analyzed by cytoflex S Flow Cytometer. Data represented the means ± the SEM (n = 3) and were tested for statistical significance using a two-tailed student’s *t-*test. *p < 0.05; **p < 0.01.

To test whether zebrafish BIRC2 has anti-apoptosis function, EPC cells transfected with p3×FLAG empty plasmid or BIRC2-FLAG were infected with *E. piscicida*, and next used for measuring the apoptosis rate by flow cytometry. At the early apoptosis stage, the apoptosis rates for cells transfected with BIRC2-FLAG had no significant change in the absence of infection, and were significantly lower than that transfected with p3×FLAG empty plasmid when the MOIs of *E. piscicida* were 5 and 10 (**Figure 3A**). At the late apoptosis stage, the apoptosis rates for cells transfected with BIRC2-FLAG had no significant change in the absence of infection and at an MOI of 5, but significantly lower than that transfected with p3×FLAG empty plasmid at an MOI of 10 (**Figure 3B**).

### Negative Regulation of Zebrafish BIRC2 on *E. piscicida* Infection

**Figure 4:**
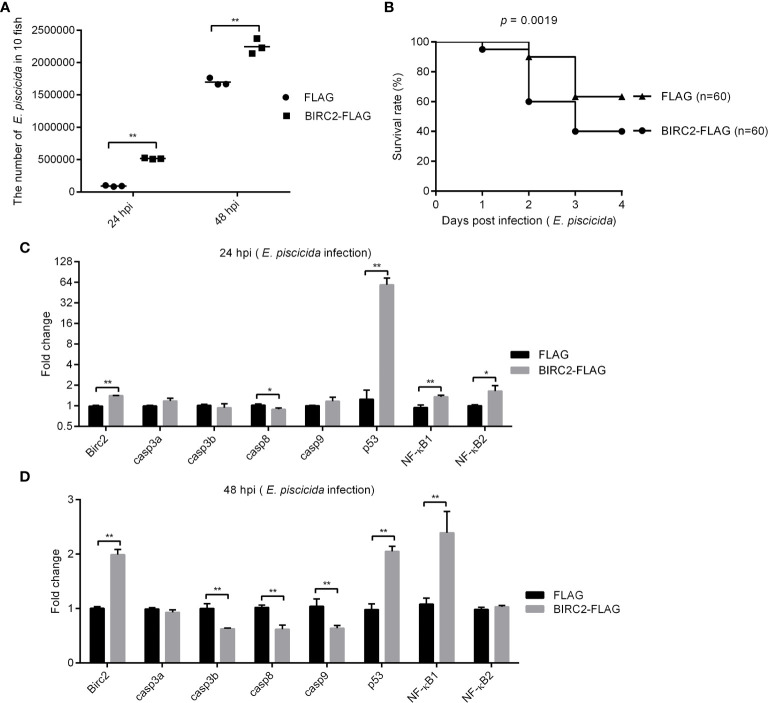
Negative regulation of zebrafish BIRC2 on *E. piscicida* infection. **(A)** The effect of zebrafish BIRC2 on the bacteria proliferation. **(B)** The effect of zebrafish BIRC2 on the larvae survival in response to *E. piscicida* infection. **(C, D)** The effect of zebrafish BIRC2 on the expression levels of apoptosis-related genes and NF-κB genes at 24 and 48 hpi. Zebrafish larvae microinjected with the p3×FLAG plasmid were used for control group. The housekeeping gene GAPDH was used for normalizing the Ct values of target genes. The fold changes relative to 1 control group were calculated using the 2^−ΔΔCt^ method. Data represented the means ± the SEM (n = 3) and were tested for statistical significance using a two-tailed student’s *t* -test. *p < 0.05; **p < 0.01.

To determine the effect of zebrafish BIRC2 in bacterial infection, zebrafish larvae overexpressed with BIRC2-FLAG or the empty plasmid were infected with *E. piscicida*. Compared with the control group transfected with the empty plasmid, bacterial load in the zebrafish larvae overexpressed with BIRC2-FLAG were higher both at 24 and 48 hpi (**Figure 4A**). The survival rate of zebrafish larvae overexpressed with BIRC2-FLAG was reduced about 23.33% (**Figure 4B**).

To determine the possible correlation between zebrafish BIRC2 and caspases or p53, we infected zebrafish larvae overexpressed with BIRC2-FLAG or the FLAG empty plasmid with *E. piscicida* or left untreated, and examined the expression levels of *casp3a*, *casp3b*, *casp8*, *casp9*, and *p53* along with the expression levels of *NF-κB1* and *NF-κB2*. Compared with the control larvae microinjected with the empty plasmid, the overexpression of zebrafish BIRC2-FLAG significantly decreased the expression of *casp8*, and increased the expressions of *p53*, *NF-κB1*, and *NF-κB2* at 24 hpi, especially for *p53* with the 47.35-fold induction (**Figure 4C**). At 48 hpi, overexpression of zebrafish BIRC2-FLAG significantly decreased the expressions of *casp3b*, *casp8*, and *casp9*, increased the expressions of *p53* and *NF-κB1*, and no significant changes for *casp3a* and *NF-κB2* (**Figure 4D**).

### Caspase Activator PAC-1 Blocks the Negative Regulation of Zebrafish BIRC2 on the *E. piscicida* Infection

**Figure 5:**
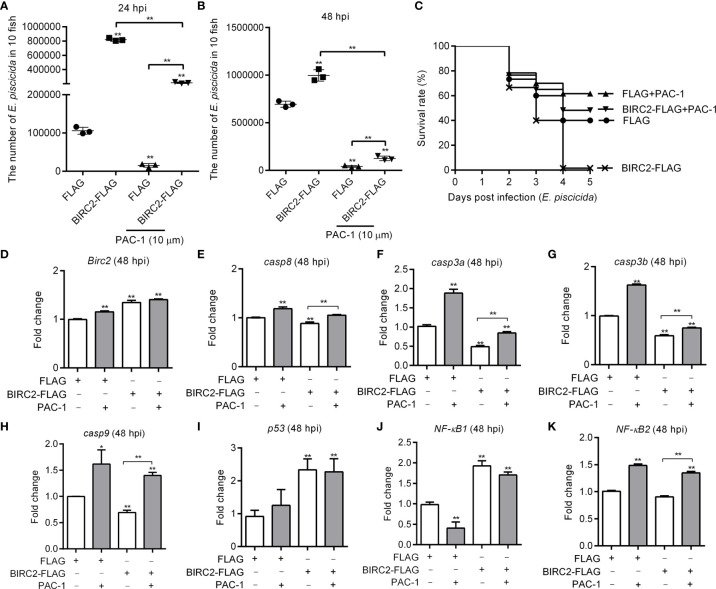
The caspase activator PAC-1 blocks the negative regulation of zebrafish BIRC2 on the *E. piscicida* infection. **(A)** PAC-1 inhibited the zebrafish BIRC2-mediated bacterial proliferation at 24 hpi. **(B)** PAC-1 blocked the zebrafish BIRC2-mediated bacterial proliferation at 48 hpi. For **(A, B)**, larvae were collected at 24 and 48 hpi, and used for colony count. Data represented the means ± the SEM (n = 3) and were tested for statistical significance using a two-tailed student’s *t* -test. **p < 0.01. The asterisk above the error bars indicates statistical significance using the group microinjected with empty plasmid and without treatment as the control group. The asterisk above the bracket indicates statistical significance between the two groups connected by the bracket. **(C)** The effect of PAC-1 on the survival rates of zebrafish larvae microinjected with p3×FLAG or BIRC2-FLAG in the case of *E. piscicida* infection. Exposures were performed in triplicate with 20 larvae per repetition (n = 60). Statistical differences using the log-rank test were observed between the FLAG/BIRC2-FLAG groups (*p <* 0.0001), FLAG/FLAG+PAC-1 groups (*p <* 0.05), and BIRC2-FLAG/BIRC2-FLAG+PAC-1 groups (*p <* 0.0001). **(D–K)** The effect of PAC-1 on the expression levels of apoptosis-related genes and NF-κB genes in zebrafish larvae microinjected with p3×FLAG or BIRC2-FLAG at 48 hpi. For **(D–K)**, larvae were collected at 48 hpi, and used for qRT-PCR. Data represented the means ± the SEM (n = 3) and were tested for statistical significance. *p < 0.05; **p < 0.01. For **(A–K)**, 4 dpf larvae microinjected with the p3×FLAG or BIRC2-FLAG were treated by adding PAC-1 for 12 h, and then infected with *E*. *piscicida*.

Since overexpression of zebrafish BIRC2 inhibited the expression levels of caspases in the case of *E. piscicida* infection, we further investigated whether the inhibition of zebrafish BIRC2 on caspases were required for the negative regulation of zebrafish BIRC2 on the *E. piscicida* infection. The facts that overexpression of zebrafish BIRC2 promoted the proliferation of *E. piscicida* in zebrafish larvae were again confirmed (**Figures 5A, B** **)**. However, treatment of zebrafish larvae microinjected with BIRC2 with the caspase activator PAC-1 significantly inhibited the zebrafish BIRC2-mediated bacterial proliferation at 24 hpi (**Figure 5A**), and completely blocked the zebrafish BIRC2-mediated bacterial proliferation at 48 hpi (**Figure 5B**). Compared with zebrafish larvae microinjected with the FLAG empty plasmid, the bacterial loads in zebrafish larvae microinjected with BIRC2 even were significantly decreased by the PAC-1 treatment (**Figure 5B**). Most of all, PAC-1 treatment tremendously improved larvae survival. All the larvae overexpressed with zebrafish BIRC2 without the PAC-1 treatment died at 4 dpi, however the survival rate of zebrafish larvae overexpressed with BIRC2-FLAG but with the PAC-1 treatment was 61.67% at 5 dpi. Compared with the control group microinjected with the FLAG empty plasmid in the absence of PAC-1 treatment, no significant difference was observed for the survival rate of zebrafish larvae overexpressed with BIRC2-FLAG and with the PAC-1 treatment (**Figure 5C**).

Next we examine whether the caspase activator PAC-1 treatment rescued the zebrafish BIRC2-mediated inhibition on caspases. In the zebrafish larvae microinjected with the FLAG empty plasmid, PAC-1 treatment induced the expressions of *BIRC2*, *casp8*, *casp3a*, *casp3b*, *casp9*, and *NF-κB2*, decreased the expression of *NF-κB1*, and no significant regulation on *p53* (**Figures 5D–K**). In the zebrafish larvae microinjected with BIRC2-FLAG, PAC-1 treatment induced the expressions of *casp8*, *casp3a*, *casp3b*, *casp9*, and *NF-κB2*, and no significant regulation on the expressions of *BIRC2*, *p53*, and *NF-κB1* (**Figures 5D–K**). Compared with the group microinjected with the FLAG empty plasmid but without the PAC-1 treatment, PAC-1 treatment had no significant regulation on *casp8* and increased the expression of *casp9* in zebrafish larvae microinjected with BIRC2-FLAG (**Figures 5E, H** **)**.

### Zebrafish BIRC2 Accumulates p53 in the Cytoplasm and the Nucleus

**Figure 6:**
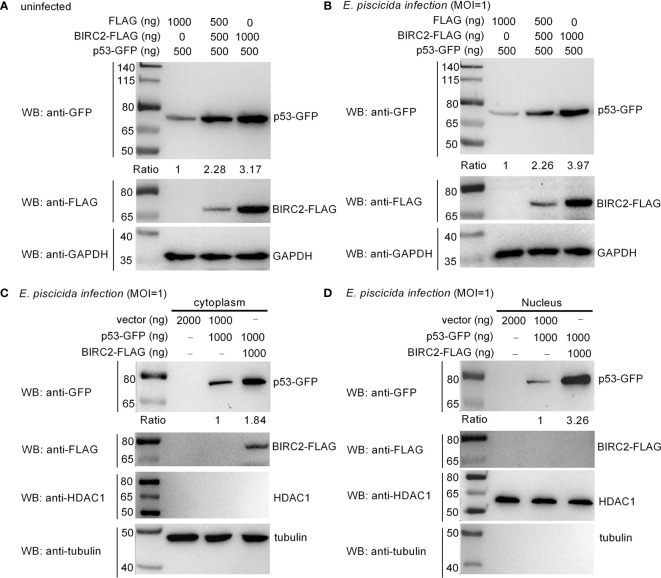
Zebrafish BIRC2 accumulates p53. **(A)** Zebrafish BIRC2 accumulates p53 in the uninfected EPC cells. **(B)** Zebrafish BIRC2 accumulates p53 in the *E. piscicida*-infected EPC cells. **(C)** Zebrafish BIRC2 accumulates p53 in the cytoplasm of infected EPC cells. **(D)** Zebrafish BIRC2 accumulates p53 in the nucleus of infected EPC cells. For **(A, B)**, EPC cells transfected with p53-GFP, p3×FLAG and/or BIRC2-FLAG were infected with *E. piscicida* for 1 h at an MOI of 1 or left untreated. At 6 hpi, the cells were washed, lysed, and subjected to Western Blotting with the indicated antibodies. For **(C, D)**, EPC cells transfected with p53-GFP, p3×FLAG and/or BIRC2-FLAG were infected with *E. piscicida* for 1 h at an MOI of 1. At 6 hpi, the cells were harvested and used for preparation of nuclear and cytoplasmic extracts. Tubulin and HDAC1 were used as loading controls for cytoplasmic and nuclear protein, respectively. The expression ratio for p53 protein was quantified by Quantity One.

Overexpression of zebrafish BIRC2 significantly increased the expressions of *p53*, especially at 24 hpi. We further investigated the effect of zebrafish BIRC2 on the protein levels of p53. In the absence of infection, the induced expressions of p53 by zebrafish BIRC2 were dependent of the dose variation (**Figure 6A**). Similar results were observed in the case of *E. piscicida* infection (**Figure 6B**). In mammals, the actions of p53 as well as its localization are not restricted to the nucleus. p53 has been found to induce apoptosis in the cytoplasm through the intrinsic mitochondria-mediated pathway (21). To examine the location of zebrafish BIRC2-mediated p53 accumulation, cytoplasmic and nuclear fractions were prepared from *E. piscicida*-infected EPC cells transfected with various indicated plasmids. p53-GFP protein was detected both in the cytoplasm and the nucleus, however BIRC2-FLAG was detected only in the cytoplasm. Furthermore, overexpression of zebrafish BIRC2 was found to accumulate p53 both in the cytoplasm and the nucleus (**Figures 6C, D** **)**.

### Zebrafish BIRC2 Colocalizes and Interacts With p53 in the Cytoplasm

**Figure 7:**
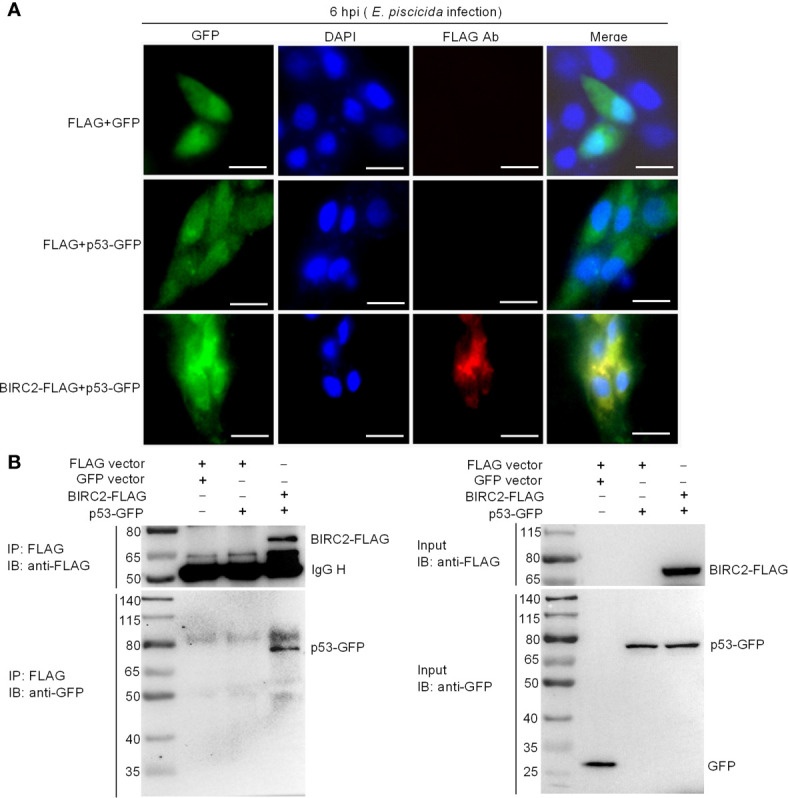
Zebrafish BIRC2 colocalizes and interacts with p53. **(A)** Immunofluorescence analysis of zebrafish BIRC2 and p53 in EPC cells. EPC cells were transfected with the indicated plasmids and then infected with *E. piscicida*. At 6 hpi, the infected cells were fixed with 4% formaldehyde for 15 min and subjected to immunofluorescence microscopy assays. Scale bar: 10 μm. **(B)** The interaction between zebrafish BIRC2 and p53. EPC cells were transfected with the indicated plasmids, then lysed and immunoprecipitated using Anti-FLAG M2-Agarose Affinity Gel beads. The input proteins and precipitates were subjected to Western Blotting using anti-FLAG and anti-pTurboGFP antibodies.

To further investigate the interactions between zebrafish BIRC2 and p53, we next conducted subcellular localization studies using transient-expression vectors to express p53-GFP and BIRC2-FLAG. Expression of the fusion proteins from these plasmids in transfected EPC cells has been confirmed by Western blotting (**Figure 6**). In control experiments, pTurboGFP fluorescence was observed over the cytoplasm and the nucleus of infected cells. p53-GFP only had a weak signal observed in the cytoplasm and the nucleus of infected cells in the absence of zebrafish BIRC2, but the obviously enhanced fluorescence signal for p53-GFP was observed in the cytoplasm of infected cells in the case of zebrafish BIRC2. Fluorescence associated with BIRC2-FLAG (Red) was only detected in the cytoplasm of infected cells. Furthermore, the merged image confirmed that zebrafish BIRC2 colocalized with p53 in the cytoplasm of infected cells, which was indicated by yellow fluorescence (**Figure 7A**).

Co-IP assays were next performed to further confirm the interaction between zebrafish BIRC2 and p53. Anti-FLAG-conjugated agarose beads were used to precipitate FLAG-containing protein complex from EPC cells. No band of p53-GFP was observed in Lane 2 (using anti-GFP antibody for IP product), which suggested that FLAG protein failed to interact with p53. However, immunoblotting analysis of anti-FLAG immunoprecipitate with an anti-GFP antibody (Lane 3) showed that the association of zebrafish BIRC2 with p53 was readily detected (**Figure 7B**).

### The p53 Inactivators Pifithrin-μ and Pifithrin-α Blocks the Negative Regulation of Zebrafish BIRC2 on the *E. piscicida* Infection

**Figure 8:**
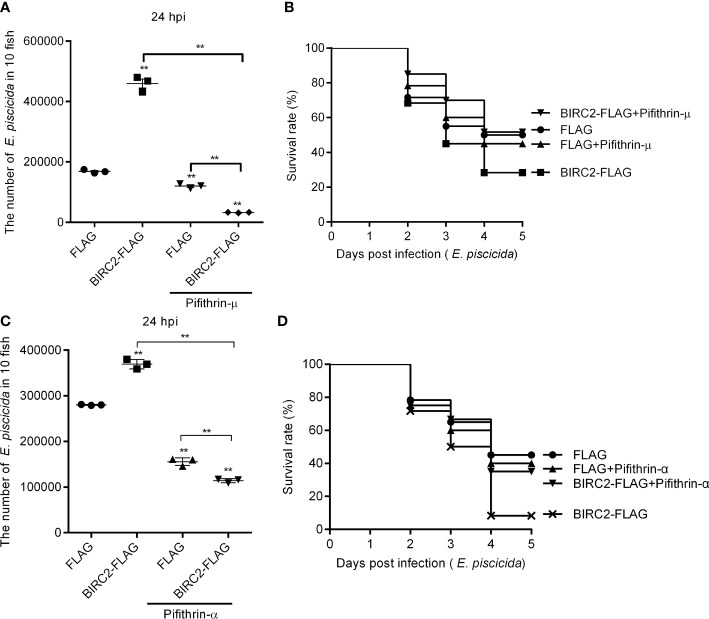
The p53 inactivators block the negative regulation of zebrafish BIRC2 on the *E. piscicida* infection. **(A)** Pifithrin-μ inhibited the zebrafish BIRC2-mediated bacterial proliferation at 24 hpi. **(B)** The effect of Pifithrin-μ on the survival rates of zebrafish larvae microinjected with p3×FLAG or BIRC2-FLAG in the case of *E. piscicida* infection. **(C)** Pifithrin-α inhibited the zebrafish BIRC2-mediated bacterial proliferation at 24 hpi. **(D)** The effect of Pifithrin-α on the survival rates of zebrafish larvae microinjected with p3×FLAG or BIRC2-FLAG in the case of *E. piscicida* infection. For **(A–D)**, 4 dpf larvae microinjected with the p3×FLAG or BIRC2-FLAG were treated by adding Pifithrin-μ or Pifithrin-α for 12 h, and then infected with *E*. *piscicida*. For **(A, C)**, larvae were collected at 24 hpi, and used for colony count. Data represented the means ± the SEM (n = 3) and were tested for statistical significance using a two-tailed student’s *t* -test. **p < 0.01. The asterisk above the error bars indicates statistical significance using the group microinjected with empty plasmid and without treatment as the control group. The asterisk above the bracket indicates statistical significance between the two groups connected by the bracket. For **(B, D)**, larvae were monitored for 5 days. Exposures were performed in triplicate with 20 larvae per repetition (n = 60). Statistical differences using the log-rank test were observed between the FLAG/BIRC2-FLAG groups (*p <* 0.05), BIRC2-FLAG/BIRC2-FLAG+ Pifithrin-μ groups (*p <* 0.01), and BIRC2-FLAG/BIRC2-FLAG+ Pifithrin-α groups (*p <* 0.01).

Since zebrafish BIRC2 was found to accumulate p53 both in the cytoplasm and the nucleus, we would like to investigate whether the induction of the cytoplasmic and nuclear p53 by zebrafish BIRC2 were required for the negative regulation of zebrafish BIRC2 on the *E. piscicida* infection. Pifithrin-μ is a cytoplasmic p53 inhibitor, which can inhibits p53 binding to mitochondria (22), however pifithrin-α is widely used as a specific inhibitor of p53 transcription activity by acting at a stage after p53 translocation to the nucleus (23, 24). Here, the p53 inactivators pifithrin-μ and pifithrin-α were used to inhibit the cytoplasmic and nuclear p53, respectively. Although treatment of pifithrin-μ inhibited the proliferation of *E. piscicida* in zebrafish larvae (**Figure 8A**), no significant differences were observed for the survival rates of zebrafish larvae overexpressed with the FLAG empty plasmid between these two groups without or with the pifithrin-μ treatment (**Figure 8B**). However, pifithrin-μ treatment significantly inhibited the proliferation of *E. piscicida* in zebrafish larvae, and completely blocked the zebrafish BIRC2-mediated bacterial proliferation at 24 hpi, when compared with zebrafish larvae overexpressed with the FLAG empty plasmid or BIRC2-FLAG in the absence of pifithrin-μ treatment (**Figure 8A**). Furthermore, pifithrin-μ treatment completely rescued larval survival for zebrafish microinjected with BIRC2 in the case of *E. piscicida* infection (**Figure 8B**). Similar results were observed for pifithrin-α treatment (**Figures 8C, D** **)**.

## Discussion

Cell death by apoptosis is a common response to microbial infections or environmental stimuli. Not surprisingly, a number of bacteria and hosts have properties that regulate apoptosis (25–27). In mammals, IAPs, caspases and p53 have been found to inhibit or promote apoptosis. A study showed that scutellarein could induce apoptotic cell death *via* inhibition of IAPs and activation of p53, which led to caspase-dependent apoptosis in gastric cancer cells (28). However, there are few reports about the functional correlations among BIRC2, caspases and p53 in pathogen infections, especially in teleost fish. Here, we have identified the conserved antiapoptosis activity of zebrafish BIRC2 and the negative regulation of zebrafish BIRC2 in bacterial infection, with the decreased expression levels of caspases and the induced expressions of p53 and NF-κB genes. Importantly, BIRC2-mediated regulation on the caspases and p53 are required to promote the proliferation of *E. piscicida* and impair larvae survival *in vivo.*

IAPs are a group of structurally related proteins initially identified to inhibit virus induced apoptosis (29). Among these IAPs, BIRC2 was firstly identified as signaling components associated with the TNF-R1 signaling complex (30). Since BIRC2/BIRC3 and TRAF2/3 could form a cytoplasmic complex, where BIRC2/BIRC3 mediated ubiquitination and degradation of TRAF3, the roles of BIRC2/BIRC3 in viral infection were extensively investigated. Many studies have suggested the inconsistent effects of mammalian BIRC2 in viral infection. It was found that knockdown of BIRC2 enhanced HIV-1 or VSV replication and abrogated poly I:C-mediated antiviral activity (31, 32), however antagonizing cIAPs and the deficiency of a liver-specific BIRC2 and total BIRC3 efficiently controlled Hepatitis B virus (HBV) infection (33, 34). The role of mammalian BIRC2 in bacterial infection was also observed. For instance, cIAP-1 knockout mice failed to clear the infection of *Chlamydophila pneumoniae* from their lungs, however a gradual reduction of bacterial load was observed for infected WT mice (35). In the present study, we observed that in zebrafish larvae infected with *E. piscicida*, BIRC2 expression was significantly induced. In addition, overexpression of BIRC2 significantly impaired larvae survival and inhibited apoptosis, which had the higher bacterial loads. The present study confirms that the antiapoptotic protein BIRC2 negatively regulates bacterial infection, which is consistent with our recent report showing that proapoptotic protein BID positively regulates bacterial infection (36). Together, these results suggest that apoptosis-related proteins may play an important role in the regulation of pathogen infections, in addition to their conserved function in apoptosis.

Cellular fate is determined by the balance between proapoptotic and prosurvival molecules. Activation of caspases is a key event for the induction of apoptosis, but antiapoptotic proteins of the IAPs family block the caspase-induced apoptosis (37, 38). Among antiapoptotic proteins, cellular BIRC2 and BIRC3 have been identified as components of a plasma membrane-bound signaling complex I containing themselves, tumor necrosis factor receptor (TNFR), RIP1, TNFR-associated factor 2 (TRAF2) and the TNFR-associated death domain (TRADD) to confer prosurvival properties (39, 40). BIRC2 and BIRC3 are found to regulate prosurvival NF-κB pathway in the non-canonical pathway by ubiquitination of NF-κB-inducing kinase (NIK) and in the canonical pathway by ubiquitinating RIP1 and enabling recruitment of IKK kinase and E3 ubiquitin ligase complex LUBAC (41–43). In zebrafish, previous study revealed that the BIRC2 null mutation resulted in embryonic lethality caused by severe hemorrhage and vascular regression due to the caspase-8-dependent apoptotic cell death (39). However in the present study, although BIRC2 overexpression inhibited apoptosis and induced the transcription levels of NF-κB genes in the case of *E. piscicida* infection, the death rate of zebrafish larvae microinjected with BIRC2 was higher than the control group. *Edwardsiella tarda*-induced inhibition of apoptosis was a strategy for intracellular bacterial survival and replication inside host cells (26), which is detrimental for the survival of host. We speculate that the present data, which are different from the previously reported prosurvival function of BIRC2, may be due to the interactive effects by *E. piscicida*-mediated apoptosis and BIRC2-mediated pathways including NF-κB, p53 and apoptotic cell death pathways.

Mammalian BIRC2 and BIRC3 can bind to caspase-3, caspase-7, and caspase-9. Although the direct physical association between zebrafish BIRC2 and caspases were not investigated in the present study, overexpression of BIRC2 with *E. piscicida* infection resulted in low-level suppression for multiple members of the caspase family. However the interference of the inhibition effects of zebrafish BIRC2 on the caspases by the caspase activator PAC-1 improved larvae survival, with the decreased bacterial loads. Furthermore, previous studies showed that caspases controlled antibacterial immune defenses *via* multiple mechanisms. For example, mice defective in caspase-8 and/or RIP3 were highly susceptible to *Yersinia pestis* infection, and caspase-8-mediated cell death overrides blockade of NF-κB and MAPK signaling by the *yersinia* virulence factor YopJ to promote anti-*yersinia* immune defense (44, 45). Caspase-8 has also been found to control cytokine expression independently of RIP3, and is necessary for host defense against pathogen infection and controlling TLR-induced cytokine production independent of cell death (46). The present data demonstrated that treatment of zebrafish larvae microinjected with BIRC2 with the caspase activator PAC-1 completely blocked the negative regulation of BIRC2 on the *E. piscicida* infection, with the reduced inhibition on the casp3 and without inhibition on casp8 and casp9. Since that the treatment of caspase activator PAC-1 conferred anti-*Edwardsiella* immune defense (**Figures 5A–C**) and that the endogenous expression of BIRC2 was induced by *E. piscicida* infection, activation of caspases could be a promising strategy to dampen the BIRC2-mediated bacteria proliferation and promote antibacterial immune defense.

The functions of p53 as a tumor suppressor are diverse, and include regulation of cell cycle arrest, induction of apoptosis, and regulation of metabolic pathways so on (47, 48). It is becoming increasingly evident that numerous bacterial pathogens have been shown to inactivate p53 to impede the immune protective response of the host. Bacteria such as *Helicobacter pylori*, *Chlamydia trachomatis*, *Shigella flexneri*, and *Neisseria gonorrhoeae* mediate protein degradation of p53 to prevent the induction of apoptosis, the production of intracellular reactive oxygen species (ROS) and other p53 regulated pathways that are detrimental for bacterial growth and dissemination (49). Cellular levels of p53 are also regulated by host factors. IKKβ can regulate p53 protein stability through the canonical NF-κB pathway (50). BIRC3 prevented p53 degradation by inhibiting IKKα/β-mediated activation of MDM2 (51). The direct correlation between antiapoptotic protein BIRC2 and p53 were confirmed in the present study. The interaction and co-localization between zebrafish BIRC2 and p53 were found in the cytoplasm of infected cells. Besides, BIRC2 overexpression markedly increased p53 levels in the case of *E. piscicida* infection, which was different from the suppression of proapoptotic protein BID on the p53 (36). Unexpectedly, zebrafish larvae overexpressed with BIRC2 presented the increased p53 levels, but resulted in an increased infection rate and bacterial growth and dissemination. The present study is different from the hypothesis that p53 has an antibacterial function (49). Most of all, the p53 inactivators had no significant effect on larval survival, but completely rescued larval survival for zebrafish microinjected with BIRC2 in the case of *E. piscicida* infection, which suggested that the accumulations of the cytoplasmic and nuclear p53 were required for the negative regulation of zebrafish BIRC2 on the *E. piscicida* infection.

In summary, our results suggest for the first time that besides their role in apoptosis regulation, zebrafish BIRC2 plays a prominent role in the negative regulation of *E. piscicida* infection by down-regulating caspases and accumulating p53. A study has shown that mammalian BIRC2 physically interacts with RIP2 through a non-CARD-CARD interaction and functions as an E3 ubiquitin ligase for RIP2 ubiquitination, which are required for innate immune signaling by the antibacterial pattern recognition receptors NOD1 and NOD2 (52). Based on this and our study, it is possible that piscine BIRC2 is involved in the regulation of immune responses mediated by NOD1 and NOD2 signaling in response to bacterial infection. Moreover, it might also be of importance to study the role (or roles) of the other apoptosis-related protein in pathogen infection.

## Data Availability Statement

The original contributions presented in the study are included in the article/supplementary material. Further inquiries can be directed to the corresponding author.

## Ethics Statement

All animal experiments were conducted in accordance with the Guiding Principles for the Care and Use of Laboratory Animals and were approved by the Institute of Hydrobiology, Chinese Academy of Sciences (Approval ID: IHB 2013724).

## Author Contributions

MC conceived and designed the experiments. LC, DY, and JX performed the experiments and analyzed the data. MC and LC wrote the manuscript. MC and HF revised the manuscript. All authors contributed to the article and approved the submitted version

## Funding

This study was financially supported by the National Key Research and Development Program of China (Grant No. 2019YFD0900703), the National Natural Science Foundation of China (Grant No. 31873046) and the Wuhan Application Foundation Frontier Project (Grant No. 2019020701011467).

## Conflict of Interest

The authors declare that the research was conducted in the absence of any commercial or financial relationships that could be construed as a potential conflict of interest.

## Publisher’s Note

All claims expressed in this article are solely those of the authors and do not necessarily represent those of their affiliated organizations, or those of the publisher, the editors and the reviewers. Any product that may be evaluated in this article, or claim that may be made by its manufacturer, is not guaranteed or endorsed by the publisher.
